# CMH-Small Molecule Docks into SIRT1, Elicits Human IPF-Lung Fibroblast Cell Death, Inhibits Ku70-deacetylation, FLIP and Experimental Pulmonary Fibrosis

**DOI:** 10.3390/biom10070997

**Published:** 2020-07-02

**Authors:** Jenya Konikov-Rozenman, Raphael Breuer, Naftali Kaminski, Shulamit B. Wallach-Dayan

**Affiliations:** 1 Lung Cellular and Molecular Biology Laboratory, Institute of Pulmonary Medicine, Hadassah–Hebrew University Medical Center, POB 12000, Jerusalem 91120, Israel; jenyak86@gmail.com (J.K.-R.); raffibreuer@gmail.com (R.B.); 2 Department of Pathology and Laboratory Medicine, 670 Albany St, 4th Floor, Boston University School of Medicine, Boston, MA 02118, USA; 3 Section of Pulmonary, Critical Care and Sleep Medicine, Department of Internal Medicine, Yale University School of Medicine, POB 208057, 300 Cedar Street TAC-441 South, New Haven, CT 06520-8057, USA; naftali.kaminski@yale.edu

**Keywords:** CMH, Ku70-SIRT1, FLIP, apoptosis, fibroblasts, IPF-resolution

## Abstract

Regenerative capacity in vital organs is limited by fibrosis propensity. Idiopathic pulmonary fibrosis (IPF), a progressive lung disease linked with aging, is a classic example. In this study, we show that in flow cytometry, immunoblots (IB) and in lung sections, FLIP levels can be regulated, in vivo and in vitro, through SIRT1 activity inhibition by CMH (4-(4-Chloro-2-methylphenoxy)-N-hydroxybutanamide), a small molecule that, as we determined here by structural biology calculations, docked into its nonhistone substrate Ku70-binding site. Ku70 immunoprecipitations and immunoblots confirmed our theory that Ku70-deacetylation, Ku70/FLIP complex, myofibroblast resistance to apoptosis, cell survival, and lung fibrosis in bleomycin-treated mice, are reduced and regulated by CMH. Thus, small molecules associated with SIRT1-mediated regulation of Ku70 deacetylation, affecting FLIP stabilization in fibrotic-lung myofibroblasts, may be a useful strategy, enabling tissue regeneration.

## 1. Introduction

Current treatments of lung fibrosis are not only ineffective, but are even shown to increase mortality in IPF [1], except Pirfenidone and Nintedanib, which prolong survival [2]. Expression of cellular FLICE inhibitory protein (FLIP) correlates with resistance to Fas- and immune cell-induced apoptosis [3], and with escape from in vivo immune surveillance [4,5,6,7]. Downregulation of c-FLIP results in loss of this resistance, in particular in fibroblasts, which are responsible for the progression of pulmonary fibrosis [3,8,9].

Sirtuins are a class of NAD^+^-dependent deacetylases with diverse cellular localization and targeting [10]. SIRT1, a class III histone deacetylase, is linked with fibrotic diseases [11,12,13]. SIRT1 is a multifunctional protein shown to be involved in fibrosis and aging of various organs [14,15], with pro-cancerous roles [16] and with particularly contradictory results in bleomycin (BLM)-induced fibrosis [17,18,19,20]. Interestingly, lysine residues of nonhistone molecules [21,22] relevant to cell survival (reviewed by: [21,22]), such as Ku70 [23], can be deacetylated by SIRT1 [24]. In fact, in cancer cells, SIRT1 inhibition augmented Ku70-acetylation, encouraging FLIP destabilization [23]. On the other hand, Ku70-deacetylation stabilized FLIP and prevented cell death [25]. 

In previous studies, we found that the Fas surface molecule is overexpressed on lung myofibroblasts from mice with bleomycin-induced fibrosis, and in humans with IPF [26]. Nevertheless, myofibroblasts from lungs with active fibrosis acquire resistance to Fas-induced apoptosis and an ‘immune-privilege-like’ phenotype, enabling their escape from immune surveillance and unremitted accumulation [27]. In addition, myofibroblasts from human and mice lungs increase FLIP levels during evolution of fibrosis [28]. We further showed that FLIP expression plays a critical role in myofibroblast proliferation and resistance to Fas-induced apoptosis [28] and that FLIP stabilization is regulated by SIRT1 deacetylation activity on Ku70 [29].

Previously, SIRT1 was associated with deacetylation of histones and downregulation of FLIP mRNA levels. Increased FLIP expression was previously detected by us and others [8,28] in fibrotic-lung myofibroblasts, in the experimental model, and in humans with IPF. In this study, we aimed to determine whether small molecule 4-(4-chloro-2-methylphenoxy)-N-hydroxybutanamide (CMH) (Cat. No. 5809354, ChemBridge Corp, San Diego, CA, USA), previously shown to downregulate expression of FLIP via HDAC3,6 and 8-mediated histone deacetylation affecting FLIP mRNA in cancer cells in vitro [30,31], functions, specifically in lung fibroblasts, as a SIRT1 inhibitor, destabilizing the FLIP and Fas signaling of apoptosis. Moreover, we aimed to assess the in vivo effect of CMH on the evolution of experimental pulmonary fibrosis. Our results clearly show inhibition of SIRT1 activity with small molecules, as CMH is a mechanism associated with FLIP stability that may pave the way for a novel therapeutic approach in lung fibrosis.

## 2. Materials and Methods 

### 2.1. Human Lung Myofibroblasts

Differentiated fibroblasts from IPF-lungs and controls from patient lung biopsies performed for tumor diagnosis were purchased from Carol Feghali-Bostwick (Medical University of South Carolina, Charleston, SC, USA). Informed consent was obtained under a protocol approved by the Institutional Review Board for Human Research at the Medical University of South Carolina. The samples were labeled and delivered to our laboratory with a code to anonymize them. LL 97A (AlMy) (ATCC^®^ CCL-191™)-IPF-lung, and LL 24 (ATCC^®^ CCL-151™)-normal lung fibroblast cell lines were used.

### 2.2. Animals

C57BL/6 and mutant mice, male, 11–12 weeks old (Harlan Sprague Dawley, Indianapolis, IN, USA) were used. All experiments involving human cells conform to the relevant regulatory standards and were approved by the Institutional Review Board of the Hadassah-Hebrew University Medical Center (Research Permission MD-15-14590-5, issued on 16.12.2015). Mice were maintained under specific pathogen-free conditions with adherence to institutional guidelines for the care and use of laboratory animals.

### 2.3. Oropharyngeal Aspiration (OA) and Induction of Lung Fibrosis in Mice

Oropharyngeal aspiration of BLM (OA-BLM) was performed as previously detailed [26]. Lung fibrosis was assessed via semi-quantitative morphological index [27]. Lung collagen was measured using a Sircol Collagen Assay kit (Biocolor, Belfast, Northern Ireland) and a standard protocol of trichrome staining.

### 2.4. Isolation of Mouse Lung Myofibroblasts 

Myofibroblast isolation and culture has been described by us in detail elsewhere [26,27].

### 2.5. Cell Death and Apoptosis

Apoptosis was assessed with Annexin V affinity labeling, trypan blue exclusion, and caspase-3 cleavage in WB, as seen in the figures and described previously [26,27,32].

### 2.6. Immunohistochemistry (IHC) Staining of Lung Tissue Sections

Performed as we previously described [26,27].

### 2.7. FLIP Protein in Lung Myofibroblasts

Standard WB and flow cytometry were performed as we described previously [33].

### 2.8. Immunoprecipitation and Immunoblotting

Standard protocols have been described in detail previously [28].

### 2.9. Docking CMH into SIRT1

AutoDock VINA v.1.1.2 was used based on human SIRT1 crystal structure from the Protein Data Bank (PDB entry 4zzj). Both ligands and water molecules in 4zzj were removed using the Discovery Studio Visualizer 4.5. Hydrogen atoms were added using the MolProbity server [34]. Kollman united atom-type charges and solvation parameters were added to the model with the aid of AutoDockTools (v. 1.5.6.; Molecular Graphics Laboratory, La Jolla, CA, USA) [35]. CMH structure was from the PubChem database. Partial charges of the ligands were assigned using the Gasteiger–Marsili method with the aid of AutoDockTools. The AutoDock VINA parameter “Exhaustiveness”, which determines how comprehensively the program searches for the lowest energy conformation, was set to a high value—18—and the size of the grid box was set as 22 Å × 16 Å × 20 Å for covering the catalytic site. Ligand rotatable torsions were released.

### 2.10. Data Analysis and Statistics

The Kruskal–Wallis test was applied to compare variables measured at different time intervals or following different treatments. The Mann–Whitney test with the Bonferroni correction was used to test for statistical significance. Two-way ANOVA was used to assess time and treatment effects and interactions. All data are presented as mean ± standard error.

## 3. Results

### 3.1. CMH Docks into SIRT1 Lysine Binding Site and Inhibits SIRT1 Activity of Ku70 Deacetylation, Destabilizes Ku70/FLIP Complex, and FLIP in IPF-Lung Myofibroblasts

Employing a high-throughput chemical screening strategy, a small molecule inhibitor of c-FLIP, 4-(4-chloro-2-methylphenoxy)-N-hydroxybutanamide (CMH) (5809354) that downregulates c-FLIP mRNA expression has been identified [36,37]. CMH but not its inactive analog 4-(4-chloro-2-methylphenoxy)-N-(3-ethoxypropyl) butanamide (CMB) (6094911) was further shown to downregulate c-FLIP^L^ and c-FLIP^S^ levels in MCF-7 cancer cells [31].

**Figure 1:**
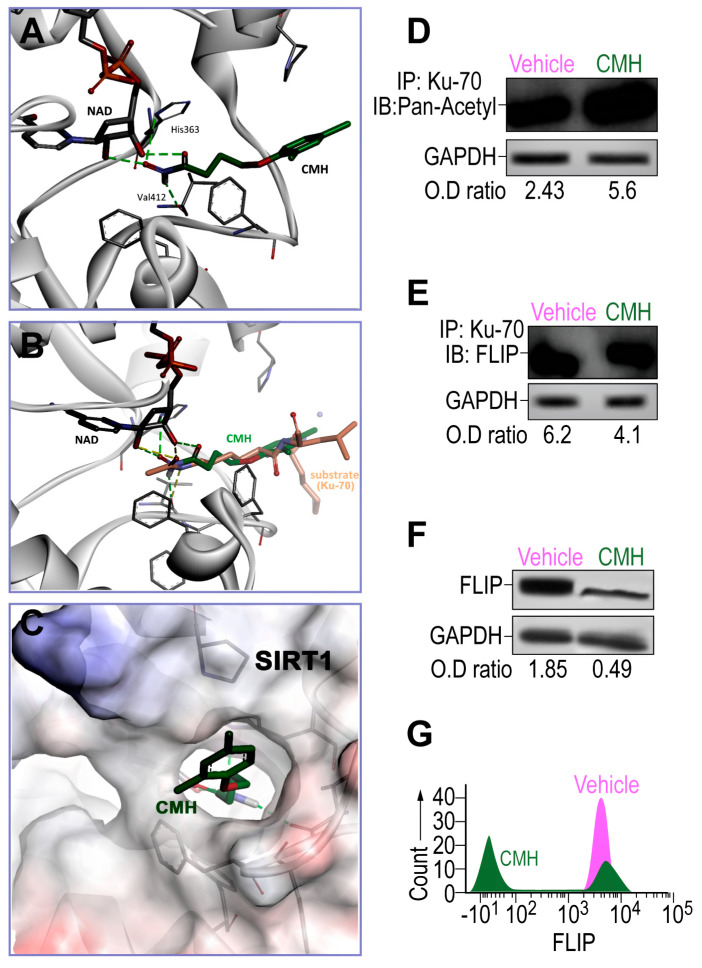
The effects of 4-(4-chloro-2-methylphenoxy)-N-hydroxybutanamide (CMH) docking into SIRT1 and CMH on FLICE inhibitory protein (FLIP) expression, Ku70 acetylation, and Ku70/FLIP complex, in human idiopathic pulmonary fibrosis (IPF)-lung myofibroblasts. Docking assessment by AutoDock VINA v.1.1.2 and Western blot or in IPF-lung myofibroblast CCL-191 cell line. (**A**) CMH docks into the SIRT1 narrow hydrophobic pocket. (**B**) Crystallographic structure superimposition of CMH and SIRT1 substrate shows high similarity to acetylated lysine. (**C**) CMH in the SIRT1active site binding pocket. WB of IP-Ku70 followed by (**D**) pan-acetyl mAb IB, or (**E**) anti-FLIP mAb of CMH (30 µM)—vs. 4% dimethyl sulfoxide (vehicle)-treated IPF-lung myofibroblast CCL-191 cell line (3 × 10^5^). (**F**) WB and, (**G**) flow cytometry analyses using anti-FLIP mAb. Representative of five experiments. *n* = 4–5. * *p* < 0.05.

As determined in silico by AutoDock VINA v.1.1.2, CMH, the pro-apoptosis molecule that downregulates FLIP in cancer cells [31,36,37] docked into a narrow hydrophobic pocket in SIRT1 (Figure 1A–C). Hydroxamate moiety created four hydrogen bonds—two between CMH and the NAD cofactor, one with the main chain carbonyl of Val412, and one between the His363 side chain of SIRT1 and CMH (Figure 1A). The predicted CMH binding mode is highly similar to the acetylated lysine substrate binding mode, as determined by superimposition of CMH in the crystallographic structure and described substrate (Figure 1B). CMH fits well in the narrow binding pocket (Figure 1C), suggesting CMH as a previously unrecognized SIRT1 inhibitor. 

Thereafter, the effects of CMH on the expression and activity of SIRT1 should be evaluated using Western blotting and activity assay in the IPF-CCL-191 cell line. CMH doses were calibrated in a way that the chosen dose does not cause toxicity on IPF- nor on normal-lung myofibroblasts (data not shown). FLIP in normal lungs had decreased by a negligible amount. Indeed, following CMH exposure (30 µM, 72 h), IPF-lung myofibroblasts although keeping stable SIRT1 expression levels (not shown), they show an affected activity of SIRT1, as indicated by the increased acetylation of Ku70, a SIRT1 nonhistone substrate [23,25,29], from an optical density ratio (OD) of 2.43 to 5.6 in pan-acetyl immunoblots (IB) of Ku70 immunoprecipitate (IP) (Figure 1D). This inhibition of SIRT1 activity detected by increased Ku70 acetylation was accompanied by a decreased formation of Ku70/FLIP complex detected in Western blot OD from 6.2 to 4.1 in FLIP-IB, constituting a reduction of 33% in the Ku70-FLIP complex (Figure 1E). FLIP decrease, from an OD of 1.85 to 0.49, was detected in WB (Figure 1F), and a 60% reduction from a mean fluorescence intensity (MFI) = 10^3^ (green) to only 10^1^ (purple) (Figure 1G) was detected with flow cytometry. This may negatively affect the capacity of myofibroblasts to survive and proliferate in pulmonary fibrosis [28]. Thus, SIRT1-mediated Ku70-deacetylation, which we have recently showed [29] to stabilize IPF-lung myofibroblast FLIP via Ku70/FLIP complex, may be inhibited by CMH. 

### 3.2. CMH Stimulates Apoptosis Pathways Regulated by SIRT1/Ku70 in Human IPF-Lung Myofibroblasts

**Figure 2:**
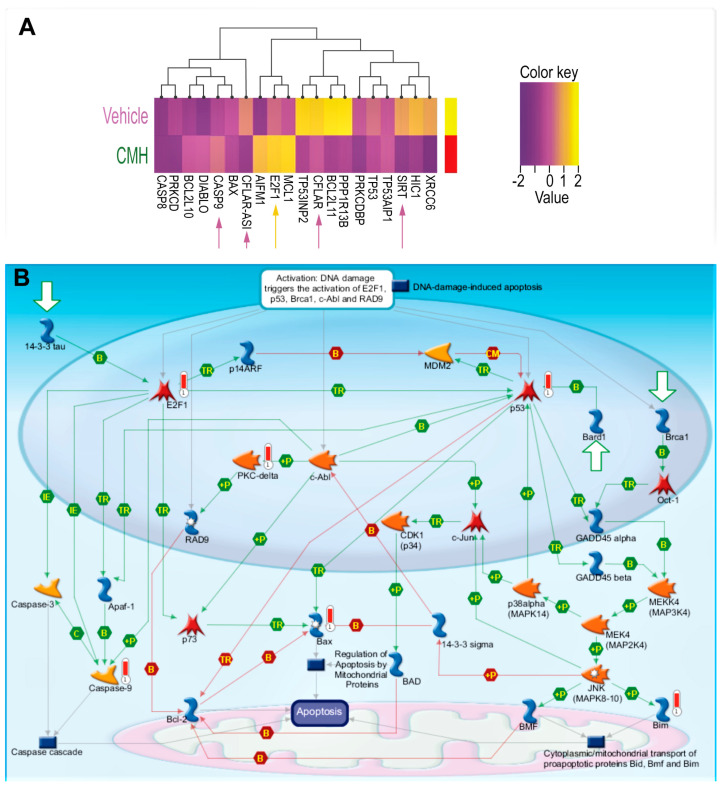
CMH decreases SIRT1-mediated survival signaling in IPF-lung myofibroblasts. RNA-seq and specific gene changes of CMH (30 µM) and fold ratio to vehicle (4% DMSO) in the IPF-lung myofibroblast CCL-191 cell line. (**A**) Heat map. K-means hierarchical clustering (K = 10) of 10,000 genes. Yellow indicates high-level expression and red-purple indicates low-level expression (Z score range −2 to +2). Both plots show two distinct clusters with significant signatures, including overexpressed genes belonging to different survival pathways (*p* = 0.0021, adjusted *p* <0.0325; *p* = 0.0045, adjusted *p* < 0.0156). (**B**) MetaCore maps’ signaling pathways explored using the systems biology tool from Genego (Genego Inc., St Joseph, MI, USA).

RNA-seq array analyses of IPF-lung fibroblasts were performed before (vehicle), and after CMH treatments (30 µM 72 h), followed by unsupervised clustering for differentially expressed genes in SIRT1-mediated signaling (Figure 2A,B, Table 1). Heat maps of gene expression normalized data, which were scaled to give all genes equal variance, showed decreases in SIRT1 and SIRT1-signaling with increments in E2F and caspase-9 following CMH exposure (Figure 2A, Table 1). A MetaCore pathway map (Genego Inc., St Joseph, MI, USA) (Figure 2B) revealed increased p53 phosphorylation by MAPk (“+P”, green arrow, right side of map), increased Bax activity (red “thermometer”), Bax-mediated apoptosis cascades, p53 phosphorylate (green arrow), and overexpressed PKC (red “thermometer”), which in turn, phosphorylate and activate RAD pathways (Figure 2B, center of map). The map clearly shows CMH-mediated upregulation of E2F1 (Figure 2B, red “thermometer”, left part of map) with downstream activation of Apaf-1 (Figure 2B, green arrows, left part of map) and upregulation of caspase-9 (Figure 2B, red “thermometer”, left part of map). 

E2F1–SIRT1 interplay bears some resemblance to that of the p53-SIRT1 axis and may be part of the mechanism controlling the fine balance between cell repair and apoptosis [38]. Of note, as detailed in our previous study [39], we did not detect the 75kDa-cleaved variant of SIRT1 in IB assays; hence, we do not expect its accumulation would affect apoptosome assembly in our cells.

### 3.3. CMH Triggers and Boosts Fas-Death Signaling in Human IPF-Lung Myofibroblasts

**Figure 3:**
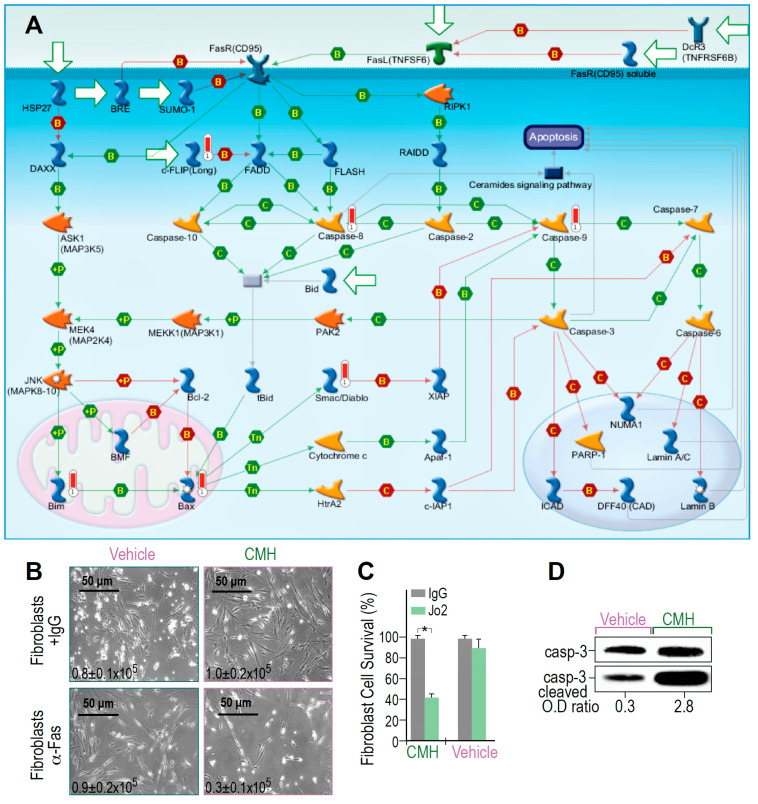
CMH increases SIRT1-regulated Fas- cell death and Fas- cascades in IPF-lung myofibroblasts. Survival pathways in RNA-seq and specific gene changes of CMH (30 µM), and fold ratio to vehicle (4% DMSO) in the IPF-lung myofibroblast CCL-191 cell line. (**A**) MetaCore Fas pathway maps (Genego Inc., St Joseph MI, USA). Cell death and Fas/FasL cascade pathway analyses; +P, green arrows—increased phosphorylation; red thermometer—increased activity and overexpression of genes. (**B**) Light microscopy images with trypan blue exclusion (inserted numbers), and (**C**) Percentage of fibroblast survival, following anti-human Fas mAb-mediated cell death, in CMH-, or control (vehicle)-treated lung fibroblasts; *n* = 4, *p* < 0.05. (**D**) Caspase-3 cleavage in WB of CMH vs. vehicle and Jo2 (20 µg, 48 h) anti-Fas mAb-treated IPF lung myofibroblasts in the CCL-191 cell line. Representative of four experiments.

MetaCore RNA-seq arrays of IPF-lung myofibroblasts revealed that molecular events activated by Fas to induce cell death are triggered and amplified by CMH (Figure 3A). The map showed positive binding interactions between Fas receptor (CD95) and its ligand (TNFSF6), as well as the FADD-caspase-8-FLASH complex (Figure 3A, green arrows). Caspase-8 activation further targets effector caspase-3 and caspase-7, a parallel extrinsic pathway, caspases 6 and 9, MAPKs, and Bim phosphorylations with binding cascades to Bax, Apaf-1, and caspase-9, allowing Bax-mediated cell death to be detected as well (Figure 3A, green arrows). Concomitantly, inhibitors of these processes, including FLIP, show negative interaction with FADD and with the inhibitors of apoptotic proteins (IAPs). The X-linked inhibitor of apoptosis protein (XIAP), in particular, showed negative interaction with caspase-9 (Figure 3A, red arrows). Moreover, CMH increased CH-11 anti-human Fas mAb-mediated IPF-myofibroblast cell death (24 h, 10 μM) above that produced by treatment with the vehicle alone (4% DMSO), as shown by microscope images and trypan blue exclusion (Figure 3B,C), with 0.9–1 × 10^5^ viable cells in control compared to only 0.3 × 10^5^ cells following CMH. In fact, CMH-treated cells were 10 times more likely to undergo Fas-induced apoptosis (2.8 compared to 0.3), as determined by the OD of cleaved to uncleaved caspase-3 ratios (Figure 3D). Of note, the absolute numbers of Caspase-3 expression are not necessarily indicative of cell survivability [40]. Thus, CMH amplifies Fas signaling cascades above those induced by Fas alone.

### 3.4. CMH Inhibits Fibrosis Evolution in BLM-Treated Mice and Ku70-Deacetylation, Ku70/FLIP Complex, FLIP Expression in Myofibroblasts Isolated From the Lungs at Day 14 Post BLM 

**Figure 4:**
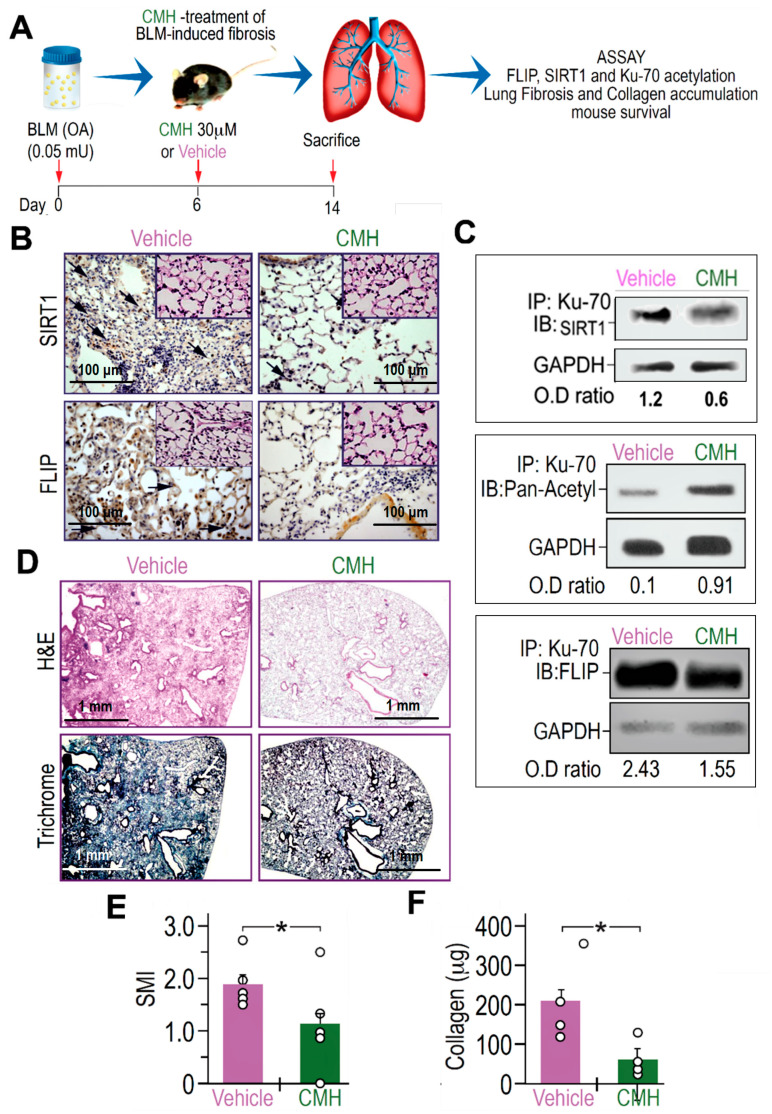
CMH downregulates FLIP, increases Ku70 acetylation, decreases Ku70/FLIP complex, and attenuates fibrosis, in bleomycin (BLM)-treated WT mouse lungs. (**A**) Schematic presentation of CMH administration into BLM-treated (0.05 mU) C57BL/6 WT mice. On day 6 after BLM, each mouse was further treated with 30 µM CMH or control 4% DMSO (vehicle) and sacrificed at day 14 post-BLM. (**B**) Immunohistochemistry (IHC) of lung SIRT1 and FLIP expression (×20 and ×40 inserts), marked by arrows in upper and lower panels, respectively. Lung fibroblast Ku70-IP and IB of (**C**) SIRT1, pan-acetyl, and FLIP (*n* = 5). (**D**) IHC of H&E and trichrome staining in lung tissue sections (upper and lower panels). (**E**) Semi-quantitative morphology index (SMI) grading lung pathology and (**F**) collagen Sircoll assay. Representative of two experiments. *n* = 4–5. * *p* < 0.02.

Increased FLIP expression was found here and previously in myofibroblasts from lungs with active fibrosis (day 14), in the experimental model, and in fibroblasts from lungs of humans with IPF [8,28]. We therefore assessed whether CMH is capable of decreasing FLIP levels and attenuate experimental lung fibrosis. To this end, on day 6 post-oropharyngeal aspiration (OA) of BLM (0.05 mU) or control saline, a single dose of CMH (30 μM) or vehicle 4% DMSO was administered into mice by a second OA (Figure 4A). As shown in [29], lung fibrosis resolution in mice correlates with loss of FLIP and of myofibroblast viability. In particular, day 6 was chosen for CMH treatment as it is removed from the initial phase of BLM-induced lung inflammation and allows enough time for the beginning of fibroblast proliferation in the lung tissue detected on day 14 (see day 14 in Figure 1A–D, respectively). The dosage was chosen out of several pre-trials as the most efficient without causing significant toxicity in this experimental setup. 

BLM-treated mice exposed to CMH vs. vehicle alone had indeed lower expression of FLIP, as well as of SIRT1, in IHC of lung tissue sections to levels detected in control saline-treated mice (Figure 4B and inserts, respectively). As we recently detected in mice with inactive SIRT1 [29], Ku70-acetylations, detected by Ku70 IP followed by pan-acetyl IB, in myofibroblasts isolated from the lungs of BLM-treated mice that were exposed to CMH, had increased from an OD of 0.1 to 0.91 (Figure 4C). Moreover, Ku70 binding to SIRT1 (Figure 4C) and complexing with FLIP (Ku70/complex), detected by Ku70 IP followed by SIRT1 or FLIP-IB respectively, had decreased from an OD of 2.43 to only 1.55 (Figure 4C).

Concomitantly, compared to vehicle, CMH-treated mice reduced hematoxylin and eosin (H&E) and collagen-trichrome staining (Figure 4D, upper and lower panels, respectively) in IHC. A semi-quantitative index (SMI) shows that CMH attenuated lung fibrosis from an index of SMI equals 2 to only 1.1 (Figure 4E), and collagen in Sircoll assay decreased from 200 µg to only 50 µg per lobe (Figure 4F). Thus, CMH shows inhibition of Ku70-deacetylation that may stabilize FLIP and Ku70/FLIP complex in lung myofibroblasts promoting fibrosis, which can be associated to inhibition of SIRT1.

## 4. Discussion

In our recent study [29], we show that FLIP decrements are related, in SIRT1-deficient fibroblasts, to their decreased complexing with Ku70, which was proven by Kerr et al. [25] to be governed by the levels of Ku70 deacetylation. In the current study, CMH is shown, for the first time, to dock into the lysine-substrate binding site on SIRT1 (Figure 1). To indicate that the CMH is an effective inhibitor of Ku70 deacetylation, we show that, compared to vehicle control, it inhibits Ku70-deacetylation (Figure 1 and Figure 4). Although direct binding assessment of CMH to SIRT1 is still required, CMH, which virtually docks to SIRT1 similar to Ku70 (Figure 1), decreases Ku70 deacetylation and FLIP levels (Figure 1 and Figure 4). CMH-mediated inhibition of Ku70 deacetylation, possibly by inhibition of the deacetylation activity of SIRT1, offers a good association to the decreased Ku70/FLIP complex detected following CMH-treatment (Figure 1). Moreover, although we have found that CMH increases histone acetylations up to 16 h of exposure of IPF fibroblasts (Supplementary Figure S1), as is the case in cancer cell by HDAC3, 6 and 8 inhibition [41], it may downregulate FLIP by inhibition of SIRT1-mediated deacetylation of nonhistone proteins as Ku70 [23], which is a key function of SIRT1 [24]. Of note, virtual binding docking assessments of CMH and FLIP have been proven to negate this option (data not shown). A thorough analysis comes up with the explanation that this is due to higher exposure of hydrophobic part to the polar edge of the protein without appropriate compensation.

CMH inhibited the binding of Ku70 to SIRT1 (Figure 4C), indicating a possible displacement of Ku70 by CMH. On the other hand, CMH effect on FLIP, via Ku70, is supported by its further disruption of the Ku70/FLIP complex and FLIP destabilization in IPF-lung myofibroblasts (Figure 1 and Figure 4). Moreover, multiple intracellular anti-apoptotic signaling pathways, known to be mediated by SIRT1 and Ku70, were altered by CMH (Figure 2), further enhancing Fas apoptosis cascades and experimental Fas-induced apoptosis in lung fibroblasts in vitro (Figure 3). In addition, CMH effects are similar to those detected by us in vivo and in vitro in mesenchymal cells [29] following specific mutation on SIRT1 gene, specifically affecting activity; the attenuation of fibroblast accumulation was associated with decreased deacetylation activity on Ku70, decreased Ku70 complexes with FLIP, and a remarkable decrease in the expression levels of FLIP (Figure 4).

CMH was docked in isolation and according to the predicted binding energy. It is not presented in relation to a drug of known activity but as an endogenous substrate (acetylated Lys) as a reference. The CMH was docked only in a catalytic site window and not as a blind dock potentially limiting the information about CMH binding to other sites on SIRT1. With this molecular modeling strategy, we simply demonstrate that CMH is capable of fitting into the SIRT1 binding site in a similar way as a substrate does. Further, NMR data to show co-crystallization of SIRT1 and CMH and mutational analyses would be of interest. A direct confirmation of interaction between CMH and the lysine binding site of SIRT1 may constitute a subject of further study, in particular with other proteins known to be deacetylated by SIRT1, such as NF-κB or FOXO1 [42]. Other members of the sirtuin family may be interesting targets for further investigation for CMH. 

We have recently found increased SIRT1 activity in fibrotic-lung myofibroblasts from IPF patients and BLM-treated mice [29]. In support of this study with CMH, the inhibition of SIRT1 deacetylation activity, as is the case in the SIRT^y/y^ chimeric mice, reduced FLIP levels, increased apoptosis cascades and decreased survival pathways in lung fibroblasts, which led to attenuation of fibrosis. Moreover, Ku70 acetylation was considerably augmented in the SIRT^y/y^ mice. Thus, although FLIP was previously demonstrated to be regulated at the transcription level by other histone deacetylases as HDAC-3, 6 and 8 [25,43] our findings support the concept of Kerr et al. that deacetylation on the nonhistone molecule, as Ku70, possibly by SIRT1, can stabilize FLIP in FLIP/Ku70 complex. 

In addition, besides having a negative feedback on itself, SIRT1 regulates several apoptosis cascades vs. survival signaling pathways, particularly by repression of p53 [44]. CMH seems to deviate the survival phenotype of IPF-fibroblasts towards apoptosis (Figure 2 and Figure 3). Indeed, interactions between E2F1 and SIRT1 regulate the apoptotic response to DNA damage by binding to each other, downregulating E2F1 expression, and inhibiting downstream Apaf1 and caspase-9 activity (Figure 2), as previously reported [45]. These findings are supported by others, who have shown that histone modifications on Fas facilitate apoptosis in fibrotic-lung myofibroblasts [46]. Histone deacetylases (HDACs) are enzymes that balance the acetylation activities of histone acetyltransferases in chromatin remodeling and proteins, and play essential roles in regulating gene transcription and protein expression [47]. Recent studies indicated that HDAC activity is also associated with the development and progression of some chronic diseases characterized by fibrosis [48], including chronic kidney disease, cardiac hypertrophy, and idiopathic pulmonary fibrosis and was suggested as a potential target for the treatment of fibrosis [12]. 

IPF lung myofibroblasts resist Fas-induced apoptosis [8]. Previously, CMH, as we show here (Figure 3), was shown to restore sensitivity to death receptor ligands in a cell-based high-throughput screening using the Fas- and TRAIL-resistant prostate cancer cell line [36,37]. In addition, a histone deacetylase (HDAC) inhibitor Vorinostat (SAHA), which was shown to downregulate lung fibrosis, potently downregulates FLIP protein expression [49]. HDAC was also shown, in vivo, to abrogate TGF-beta1-induced fibroblast–myofibroblast differentiation [50] and to restore surfactant protein-C expression in alveolar-epithelial type II cells to attenuate bleomycin-induced pulmonary fibrosis [11]. Moreover, an HDAC6-specific inhibitor Tubacin recapitulated the effects of SAHA, suggesting that HDAC6 is a key regulator of Ku70 acetylation and FLIP protein stability [25]. Small molecule CMH (4-(4-chloro-2-methylphenoxy)-N-hydroxybutanamide) was previously shown to downregulate FLIP, rendering cancer cells susceptible to apoptosis via inhibition of the class II deacetylase HDAC-8 [30,31]. Lowering anti-apoptotic signals in IPF-lung myofibroblasts may be a useful strategy to enable recovery following fibrotic injury [27]. FLIP is an anti-apoptotic molecule, and in fibroblasts, it diverts Fas-induced apoptosis towards proliferation [28]. Kim et al. thoroughly describe the logics and mechanisms of FLIP regulation by SIRT1 in hepatoma cells and the Ku70 acetylation and modulation of signaling in particular [23]. 

## 5. Conclusions

We demonstrate that inhibition of SIRT1 is associated with increased Ku70 acetylation, FLIP destabilization, neutralizing resistance to apoptosis in human IPF-lung fibroblasts and enhances a variety of apoptosis cascades. This pathway may be used to modulate fibrosis evolution in the lungs of humans with IPF. Ku70 acetylation induced by CMH may provide new therapeutic approaches to combat IPF.

## 6. Patents

The findings described in this article are protected in a provisional patent USP No. 62/897,332.

## Figures and Tables

**Table 1:** Expression of specific genes in IPF myofibroblasts of CCL-191 line incubated with CMH and the vehicle, correspondingly, calculated by 2^−∆∆Ct^, the data are represented as mean ± standard error of the mean.

Gene	Vehicle; M ± m	CMH; M ± m
XRCC6	192.46 ± 1.92	84.85 ± 22.88
HIC1	5.55 ± 0.6	0.68 ± 0.44
SIRT1	3.47 ± 0.1	0.48 ± 0.04
TP53AIP1	1.67 ± 0.22	1.11 ± 0.2
TP53	8.14 ± 1.32	1.8 ± 1.19
PRKCDBP	107.67 ± 31.83	21.87 ± 9.21
PPP1R13B	1.72 ± 1.19	0.1 ± 0.05
BCL2L11	3.28 ± 1.36	0.58 ± 0.09
CFLAR	7.36 ± 2.29	3.5 ± 1.42
TP53INP2	12.53 ± 1.61	8.16 ± 0.54
MCL1	52.13 ± 4.05	67.57 ± 34.04
E2F1	0.21 ± 0.12	0.39 ± 0.21
AIFM1	10.92 ± 2.11	12.3 ± 2.89
CFLAR-AS1	0.09 ± 0.03	0.11 ± 0.04
BAX	196.43 ± 24.62	319.29 ± 78.06
CASP9	0.38 ± 0.24	9.57 ± 6.69
DIABLO	3.32 ± 1.79	13.48 ± 3.27
BCL2L10	0 ± 0	0.13 ± 0.05
PRKCD	6.51 ± 0.7	9 ± 4
CASP8	5.4 ± 3.16	7.13 ± 4.18

## Supplementary Materials

The following are available online at https://www.mdpi.com/2218-273X/10/7/997/s1, Figure S1: Increased histone acetylation in CMH -treated IPF-lung myofibroblasts.
