# Supplementary material for: CMH-Small Molecule Docks into SIRT1, Elicits Human IPF-Lung Fibroblast Cell Death, Inhibits Ku70-deacetylation, FLIP and Experimental Pulmonary Fibrosis
Source: Biomolecules. 2020 Jul 2;10(7):997. doi: 10.3390/biom10070997 (PMC7408087; doi:10.3390/biom10070997)
Supplement: Supplementary file 1 [file biomolecules-10-00997-s001.pdf]

Biomolecules-757609
Supplementary Figure

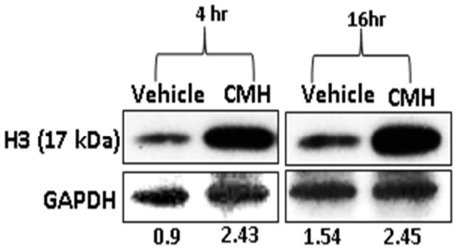

Supplementary Figure 1. Increased histone acetylation in CMH -treated IPF-lung myofibroblasts Effects of exposure to CMH for 2 different time periods on acetylation of histone-3 (H3) in human IPF-lung myofibroblasts. Myofibroblasts were incubated for 4 or 16 hr with 30 µM CMH. Total cell extract was isolated by scraping cells into SDS-lysis buffer. Protein concentrations were determined by Bradford. Equal amounts of proteins were subjected to Wb anti-acetylated protein mAb. Histone acetylation was assessed by comparing the OD ratio for CMH vs. control vehicle-treated cells. Representative of 4 experiments with similar results.
